# Comprehensive microRNA profiling of prostate cancer cells after ionizing radiation treatment

**DOI:** 10.3892/or.2014.2988

**Published:** 2014-01-21

**Authors:** CHUNG-MAN LEUNG, SUNG-CHOU LI, TING-WEN CHEN, MENG-RU HO, LING-YUEH HU, WEN-SHAN LIU, TONY T. WU, PING-CHI HSU, HONG-TAI CHANG, KUO-WANG TSAI

**Affiliations:** 1 Department of Radiation Oncology, Kaohsiung Veterans General Hospital, Kaohsiung, Taiwan, R.O.C.; 2 Department of Safety, Health and Environmental Engineering, National Kaohsiung First University of Science and Technology, Kaohsiung, Taiwan, R.O.C.; 3 Genomics and Proteomics Core Laboratory, Department of Medical Research, Kaohsiung Chang Gung Memorial Hospital and Chang Gung University College of Medicine, Kaohsiung, Taiwan, R.O.C.; 4 Molecular Medicine Research Center, Chang Gung University, Taoyuan, Taiwan, R.O.C.; 5 Bioinformatics Center, Chang Gung University, Taoyuan, Taiwan, R.O.C.; 6 Genomics Research Center, Academia Sinica, Taipei, Taiwan, R.O.C.; 7 Institute of Biomedical Sciences, Academia Sinica, Taipei, Taiwan, R.O.C.; 8 Department of Surgery, Kaohsiung Veterans General Hospital, Kaohsiung, Taiwan, R.O.C.; 9 School of Medicine, Yang-Ming University, Taipei, Taiwan, R.O.C.; 10 Department of Emergency, Kaohsiung Veterans General Hospital, Kaohsiung, Taiwan, R.O.C.; 11 Department of Medical Education and Research, Kaohsiung Veterans General Hospital, Kaohsiung, Taiwan, R.O.C.

**Keywords:** microRNA, prostate cancer, next-generation sequencing, pathway enrichment analysis, radiation, The Cancer Genome Atlas

## Abstract

MicroRNAs (miRNAs) are small, non-coding RNAs that negatively regulate gene expression and have emerged as potential biomarkers in radiation response to human cancer. Only a few miRNAs have been identified in radiation response to prostate cancer and the involvement of the radiation-associated miRNA machinery in the response of prostate cancer cells to radiation is not thoroughly understood. Therefore, the purpose of the present study was to comprehensively investigate the expression levels, arm selection preference and isomiRs of radiation-response miRNAs in radiation-treated PC3 cells using a next-generation sequencing (NGS) approach. Our data revealed that the arm selection preference and 3′ modification of miRNAs may be altered in prostate cancer after radiation exposure. In addition, the proportion of AA dinucleotide modifications at the end of the read gradually increased in a time-dependent manner after PC3 radiation treatment. We also identified 6 miRNAs whose expression increased and 16 miRNAs whose expression decreased after exposure to 10 Gy of radiation. A pathway enrichment analysis revealed that the target genes of these radiation-induced miRNAs significantly co-modulated the radiation response pathway, including the mitogen-activated protein kinase (MAPK), Wnt, transforming growth factor-β (TGF-β) and ErbB signaling pathways. Furthermore, analysis of The Cancer Genome Atlas (TCGA) database revealed that the expression of these radiation-induced miRNAs was frequently dysregulated in prostate cancer. Our study identified radiation-induced miRNA candidates which may contribute to radiosensitivity and can be used as biomarkers for radiotherapy.

## Introduction

Prostate carcinoma is the most frequently diagnosed visceral cancer in men worldwide. An increasing prevalence has been reported in recent decades (1). Radiation therapy is one of the primary modalities in prostate cancer treatment. Ionizing radiation damages cells through free radicals from the radiolysis of water that cause DNA double-strand breaks. However, the efficacy of the radiotherapy may be affected by the cellular response to radiation. Radiotherapy is highly effective in treating radiosensitive tumors and enhancing the therapeutic efficacy can increase the overall survival rate. However, the presence of radioresistant tumors leads to cancer relapse and metastasis. Understanding the tumor-radiation-related genes to predict the tumor response to radiotherapy may potentially modulate the treatment outcome for prostate cancer patients.

MicroRNAs (miRNAs) are a family of small, non-coding, single-stranded RNAs composed of ~22 nucleotides (nt) that negatively regulate protein expression at the post-transcriptional level (2). They function as gene regulators by binding to partially complementary sites of mRNAs and cause translation inhibition or direct degradation of the target mRNA. It has been suggested that miRNAs are responsible for controlling ~50% of all protein-coding genes (3). The widespread regulation of protein levels has been studied in cellular models (4). Previous studies have demonstrated that the expression of miRNAs is clearly involved in cancer development, and the deregulation of several miRNAs has been observed in various types of cancer, including prostate cancer. Porkka *et al* (5) was the first to identify a miRNA signature specific for prostate cancer by systematically profiling prostate cancer cell lines. Numerous studies have identified many dysfunctional miRNAs by using a high-throughput approach, which contributed to prostate cancer progression, including the let-7 family, miR-1, -20a, -21,-34a, -106b, -125b, -205 and -521 (6–13). Although several studies have investigated the role of these dysfunctional miRNAs to develop prostate cancer therapy, few studies have determined the roles of miRNAs in radiation response in prostate cancer. The upregulation of miR-521 reduces the response to radiation damage by specifically targeting a DNA repair protein, the Cockayne syndrome protein A (13). Li *et al* (14) found that miR-106b was dysregulated after radiation treatment and suppressed radiation-induced p21 activation, suggesting it may override radiation-induced cell cycle arrest and cell growth inhibition. Radiation delivered in daily fractions altered a greater number of miRNAs compared with single-dose radiation, and involved the upregulation of miR-34a and let-7 miRNAs (15).

Next-generation sequencing (NGS) is a high-throughput screening technology, and NGS data can be applied in investigating miRNA expression, miRNA isoforms (isomiRs) and the arm selection preferences of miRNAs. Therefore, the purpose of the present study was to comprehensively investigate the distribution of miRNAs after radiation treatment in PC3 cells by using an NGS approach. Furthermore, we explored the function of radiation-associated miRNA by conducting an *in silico* analysis.

## Materials and methods

### Cell culture and radiation treatment

A PC3 cell line was obtained from the American Type Culture Collection and was maintained in RPMI-1640 and supplemented with 10% inactivated fetal bovine serum (FBS; Invitrogen, Carlsbad, CA, USA). The cells were exposed to various radiation dosages (0, 2, 6, 10, 14 and 18 Gy) and were subsequently cultured in fresh medium. The total RNA was obtained at various time points (0, 5, 15 and 40 h after treatment) by using TRIzol (Invitrogen) according to the manufacturer’s instructions. The concentration, purity and amount of total RNA were determined using a NanoDrop 1000 spectrophotometer (NanoDrop Technologies, Inc., USA).

### Collection and preprocessing of sequence reads

PC3 cells were exposed to 10 Gy of radiation. After radiation treatment, the cells were lysed at various time points (0, 5, 15 and 40 h) for RNA extraction. The RNA samples were prepared using an Illumina small RNA preparation kit, and were subsequently sequenced using the Illumina HiSeq platform. The generated sequence reads were first subjected to quality control to remove low-quality reads. The sequence reads were then subjected to 3′ adaptor trimming to generate clean reads, as previously described (15,16). To attain a high confidence level, only the clean reads with a read count ≥2 and with a length ranging from 15 to 27 nt were included in further analyses.

### Mapping clean reads to pre-miRNAs

To investigate miRNA expression profiles in different libraries, we mapped the qualified clean reads back to human pre-miRNAs (miRBase 19). To eliminate ambiguous multiple hits during the mapping procedure, no mismatch was allowed. Previous studies reported that, when mapped back to pre-miRNAs, sequence reads usually carried mismatches preferentially located at their terminal 3′ ends (17–20). This mismatch was named the 3′ end modification. To determine whether the 3′ end modification patterns differed among libraries, as described in our previous studies (21), we trimmed and collected the terminal 3′ end mismatches one by one. In addition, the remaining perfect match reads had to be at least 18 nt in length. As a result, we kept reads with no less than 18-nt perfect alignment and 3′ end modification patterns.

### Classifying non-miRNA reads into different data sets

The sequence reads that may not be mapped back to pre-miRNAs were classified into classes by mapping to acquire different data sets with Bowtie (22) and allowing a single nucleotide variation. The sequences of mRNAs and other ncRNAs were derived from the NCBI RefSeq 47 (23). The tRNA sequences were downloaded from the Genomic tRNA database (24) and the rRNA sequences were downloaded from the SILVA database (25). The snoRNA, scaRNA and snRNA sequences were all downloaded from NONCODE (26). The sequence reads not belonging to any of the described RNA classes were uploaded to the RepeatMasker to identify repeat elements, which were classified as unknown.

### miRNA expression level according to The Cancer Genome Atlas (TCGA) data

TCGA project collects both cancer and corresponding normal tissues from hundreds of prostate cancer patients. We downloaded all level-3 miRNA expression data of prostate adenocarcinoma from the TCGA Data Portal (https://tcga-data.nci.nih.gov/tcga/dataAccessMatrix.htm). These level-3 data included calculated expressions for each miRNA derived from the Illumina HiSeq sequencing results. A total of 198 tumor samples and 50 normal samples were found at the time the data were downloaded. We kept only the expression data of 50 participants who had both miRNA expression levels from both tumor and normal tissues. Normalized quantification expression levels for these 50 participants were further examined for each investigated miRNA.

### Pathway enrichment analysis

We attempted to determine the functions of the miRNA target genes by investigating the pathways with which the miRNA target genes were involved. Therefore, we first downloaded the target genes of differentially expressed miRNAs from TargetScan 6.0, and then mapped the target genes onto the Kyoto Encyclopedia of Genes and Genomes (KEGG) pathways based on the Enzyme Commission (EC) numbers by using the R package SubPathwayMiner v.3.1 (27). Subsequently, the hypergeometric test was performed to identify significantly enriched pathways and calculate the false positive discovery rate in the FDR-corrected q-value.

## Results

### miRNA profiling of radiation-treated prostate cancer cells

**Figure 1:**
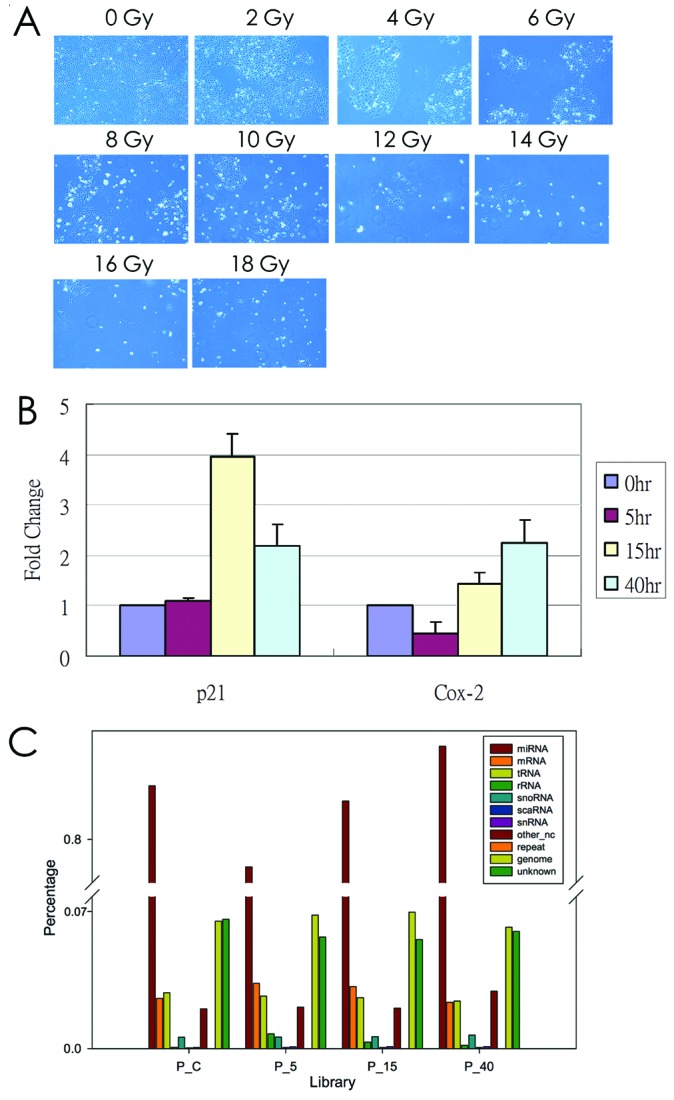
Radiation treatment of human prostate cancer cells, PC3. (A) PC3 cells were treated with various radiation doses (0, 2, 4, 6, 8, 10, 12, 14, 16 and 18 Gy) and were subsequently subjected to fresh culture medium. After culturing for an additional 4 days, the morphology was observed using light microscopy (x40 magnification). (B) The expression pattern of COX-2 and p21 in radiation-treated PC3 cells was examined using a real-time PCR method. S26 was used as an internal control. (C) The distribution of small RNA reads in 11 categories was classified.

To characterize the mechanism involved in the radiation response of prostate cancer, we used NGS to comprehensively analyze the distribution of miRNAs after radiation treatment in PC3 cells. As indicated in Fig. 1A, PC3 cells were exposed to various dosages of radiation (0, 2, 4, 6, 8, 10, 12, 14, 16 and 18 Gy) and then subjected to a fresh culture medium for an additional 4 days. We found that the growth of the PC3 cells obviously decreased when exposed to 10 Gy of radiation. Therefore, we collected cell RNA at various times (0, 5, 15 and 40 h) following the 10-Gy radiation treatment. We confirmed the expression levels of Cox-2 and p21, which may be induced by radiation at 24 h according to previous studies (16,28). The expression levels of Cox-2 and p21 may be upregulated by radiation treatment in PC3 cells (Fig. 1B). We then performed the comprehensive miRNA profile at various time-points in radiation-treated PC3 cells by using the Illumina HiSeq platform.

### Analysis of miRNA sequence reads

Once the samples were sequenced, we collected >9 million clean reads in all libraries (Table I). In addition to miRNA, we also determined which molecules were the remaining non-miRNA reads. By mapping the non-miRNA reads back to a different data set, we classified the reads into 11 categories. Fig. 1C demonstrates that miRNA accounted for 80% of all clean reads in the prostate cell libraries. Other categories accounted for relatively low proportions, which indicated the high performance of the sample preparation protocol. In addition, the proportions of the categories were considerably similar among libraries, indicating that radiation treatment did not alter the composition of RNA samples in the prostate cell libraries.

**Figure 2:**
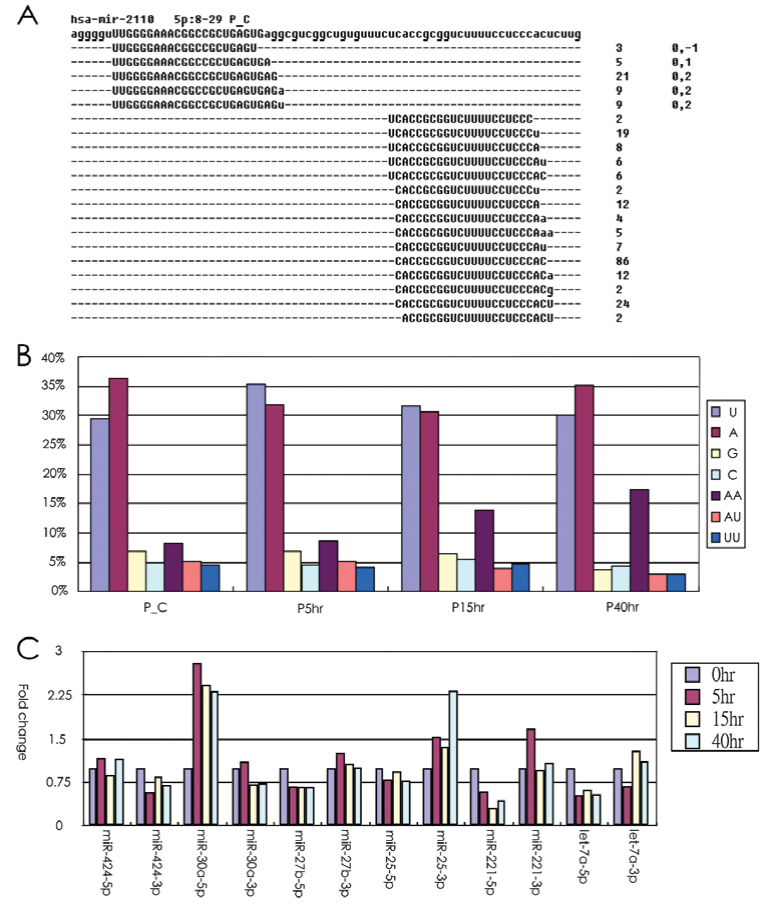
The distribution of isomiRs and -5p/-3p arm selection in PC3 radiation treatment. (A) Mapping results of hsa-miR-2110. As shown in 5p:8–29, hsa-miR-2110 encodes mature miRNA at only its 5 p arm, and miRNA spans from nucleotide 8 to nucleotide 29 of the hairpin. The integer values on the left denote the read count of each isomiR. The comma-separated values denote the position shift in the isomiR relative to the miRBase annotated positions (8 to 29). The nucleotides in lowercase type denote the sequence fragments originating from the 3′ modification event. (B) The proportion of 3′ modifications at end of reads at different time-points after PC3 radiation treatment as observed from NGS data. (C) Fold-change of the -5p/-3p arm of miRNA at different time points after PC3 radiation treatment as observed from NGS data. isomiRs, miRNA isoforms; miRNA, microRNA; NGS, next-generation sequencing.

After mapping the clean reads to the genome, most of the miRNA reads tend to exist as isomiR. As demonstrated in Fig. 2A, hsa-miR-2110-5p had 5 isomers, whereas the opposite-arm miRNA-3p had 15 isomiRs, which demonstrated that abundant miRNAs tend to have more isomiRs. Our data revealed that the isomiR quantity was highly correlated with miRNA abundance (Pearson’s correlation coefficient, 0.91). In addition, we observed that the modified nucleotides were preferentially located at the 3′ end of the sequence read (presented in lower case in Fig. 2A). The data indicated that one A nucleotide or one U nucleotide was frequently added at the end of the read. Notably, we found that the proportion of AA dinucleotides modified at the end of the read was gradually increased in a time-dependent manner after the PC3 cells were treated with radiation, which indicated that the 3′ end modification may be altered by radiation treatment in PC3 cells. Our previous studies indicated that the use of miR-5p and -3p may be altered in human cancer (29–31). In the present study, our data indicated that arm selection preference was consistent across nearly all libraries. Only a few cases were observed in which the use of -5p and -3p arm selection had different preferences at various time-points after radiation treatment (Fig. 2C). Further research is required to support these findings.

### Radiation-response miRNAs in prostate cancer

**Figure 3:**
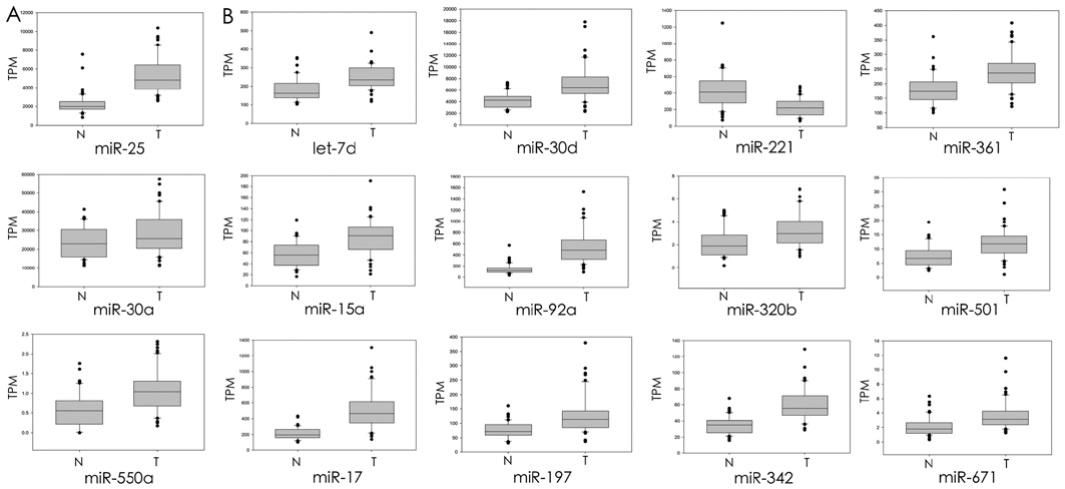
(A) Expression levels of radiation-induced miRNAs in prostate cancer. (B) Expression levels of radiation-suppressed miRNAs in prostate cancer. Expression levels of miRNAs between tumor and corresponding normal tissues from 50 prostate cancer patients were analyzed using TCGA dataset. The expression levels of miRNAs were presented in transcript per million (TPM). The expression level between tumor and normal cells was evaluated by conducting paired t-tests (P<0.05 was considered significant; NS, non-significant. ^*^P<0.05, ^**^P<0.01, ^***^P<0.001). TCGA, The Cancer Genome Atlas.

By summarizing the read count of all the isomiRs that belonged to the same mature miRNAs, we quantified the miRNA expression abundances, and presented the result in transcript per million (TPM). Twenty-two miRNAs were selected and are presented in Table II, demonstrating that their expression levels were altered >2-fold after being subjected to radiation exposure (expression of 6 miRNAs increased, and expression of 16 miRNAs decreased). To explore the putative role contributing to prostate cancer progression, we examined the effect of expression levels of radiation-associated miRNAs on prostate cancer from the available TCGA dataset by using an *in silico* analysis. We downloaded 100 miRNA expression profiles from 50 prostate cancer patients, including 50 cancer lesion and 50 corresponding normal tissues. As demonstrated in Fig. 3A, the expression levels of radiation-induced miRNAs, miR-25, miR-30a and miR-550a, were significantly upregulated in the prostate cancer cells compared with the corresponding normal tissue cells. Twelve radiation-suppressed miRNAs were identified, i.e. let-7d, miR-15a, miR-17, miR-30d, miR-92a, miR-197, miR-221, miR-320b, miR-342, miR-361, miR-501 and miR-671, and a significantly different expression between prostate cancer and the corresponding adjacent part was found, including 11 upregulated and 1 downregulated (Fig. 3B). Overall, the data indicated that most of the radiation-response miRNAs were identified as dysregulated in prostate cancer according to an *in silico* analysis (15/22; 1 downregulated, 14 upregulated and the rest demonstrated no change in expression in prostate cancer).

### Pathway enrichment analysis of miRNAs

**Figure 4:**
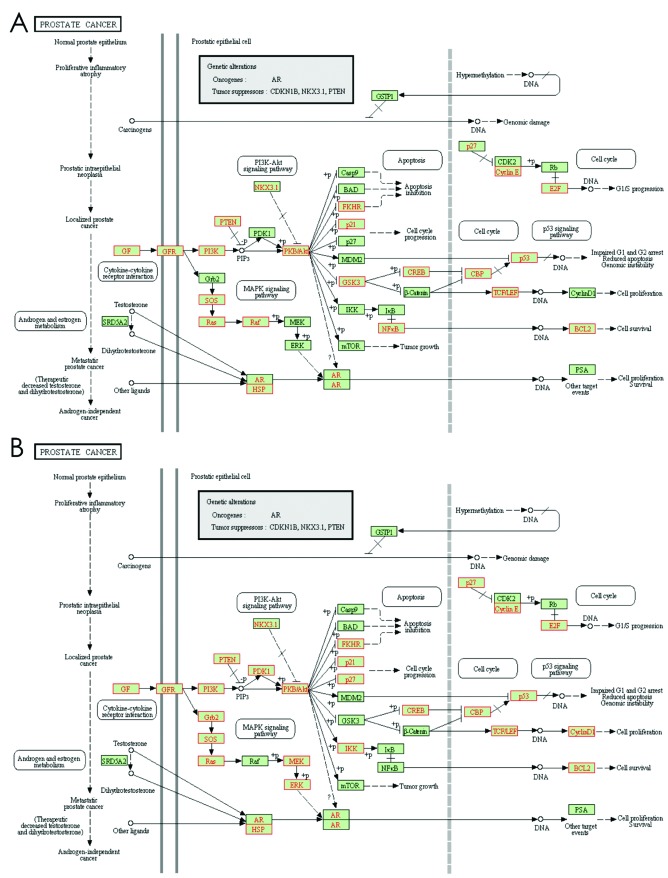
The enriched pathway of the target gene union of radiation-response miRNAs. (A) The target gene union of radiation-upregulated miRNAs enriched in the prostate cancer pathway (*FDR*=0.001). (B) The target gene union of radiation-downregulated miRNAs enriched in the prostate cancer pathway (*FDR*=3.6E-9). The target genes are labeled in red.

miRNAs can function as either oncogenes or tumor suppressors depending on their target genes. Therefore, identifying a target can facilitate elucidating the role of miRNAs in prostate cancer treatment radiation (32,33). Typically, one miRNA tends to have hundreds of target genes and a group of miRNAs co-modulated as a biological function involved in the regulation of a signaling pathway. Therefore, we further explored the biological function of radiation-response miRNAs by conducting a pathway-enrichment analysis. The putative target genes of miRNAs were obtained from TargetScan 6.0; subsequently, these target genes of the individual miRNAs were mapped onto KEGG pathways. Our data indicated that the target genes of radiation-response miRNAs were frequently significantly enriched in several cancer- or radiation-related pathways, including the mitogen-activated protein kinase (MAPK), ErbB, p53, Wnt, transforming growth factor-β (TGF-β) and mTOR signaling pathways with an *FDR* <0.05 (Table III). We also subjected the target genes of the 2-gene set, upregulated miRNA and downregulated miRNAs, to pathway-enrichment analysis. Similar results were observed; their targets were significantly enriched in the prostate cancer pathway (Fig. 4) and radiation-related pathways, including the MAPK, ErbB, Wnt and TGF-β signaling pathways (Tables IV and V).

## Discussion

Our previous studies indicated that the distributions of 3′ end modifications and the arm selection preference of miRNAs were different between normal and tumor tissues (29–31). The -5p and -3p of miRNA play a distant role by suppressing the different target genes. It was previously reported that, in contrast to the oncogenic effect of miR-17 (-5p), miR-17^*^(-3P) plays a tumor suppressive role in prostate cancer (9,34,35). The miR-28-5p and miR-28-3p also play opposite roles in colon cancer cell proliferation and migration (36). In the present study, our data showed that the -5p and -3p of particular miRNAs were differently regulated by radiation (shown in Fig. 2C). Several studies have demonstrated that miRNAs contain various ends, which were caused by either RNA editing or non-template nucleotide additions (17,18,20). These miRNA isoforms (isomiRs) contribute to increased miRNA stability or strengthened miRNA-target gene interaction and are differentially expressed in different cellular conditions, including cancer (16,37,38). Our data revealed that the proportion of AA dinucleotide modifications at the end of the read gradually increased in a time-dependent manner after the PC3 cells were treated with radiation, suggesting that radiation may influence the particular miRNA stability or efficiency of silencing targets by regulating the 3′ end modifications, which warrants further research.

miRNAs are known to function as gene silencers and are involved in modulating biological functions, including cell growth, apoptosis, the cell cycle and the metastasis of cancer (39). Comprehensive miRNA profiling of prostate cancer has indicated that several miRNAs are differentially expressed between prostate cancer and the adjacent normal, which contributes to prostate cancer progression (40–42). In the present study, we analyzed miRNA expression from TCGA database and found that the expression levels of radiation-induced miRNAs were frequently dysregulated in prostate cancer (Fig. 3). Our results are consistent with those of previous studies and demonstrated that miR-25, miR-17, miR-30d and miR-92a are overexpressed, and miR-221 is downregulated in prostate cancer (9,42–44). However, the expression levels of let-7d and miR-15a decreased according to TCGA, which contradicted the results of previous studies (45–47). These dysfunctional miRNAs have potential to be used as biomarkers for prostate cancer prognosis or diagnosis. Therefore, understanding the function of miRNAs may provide practical benefits for clinical applications. Predicting the outcome of cancer treatment is the most promising application of miRNAs. Gonzales *et al* found miR-141 to be consistent with changes in other conventional biomarkers and to the clinical outcomes, suggesting that miR-141 can be used as a marker for monitoring therapeutic response in prostate cancer patients (48). The prognostic value of miRNA expression profiling in prostate cancer has also been demonstrated by Hulf *et al*. They demonstrated that DNA methylation and histone H3K9-deacetylation of the miR-205 locus is associated with miRNA silencing and deregulation of MED1, which is predictive of a poor prognosis in localized prostate cancer (49).

Ionizing radiation is one of the 3 primary modalities used in cancer therapy. Radiation induces considerable DNA damages, which, if not repaired, cause cancer cells to progress to apoptosis and cell cycle arrest. Some cancer cells are resistant to radiation treatment due to activation of complex signaling pathways that counteract these damages, including ErbB, nuclear factor κB (NFκB), MAPK, PI3K/AKT and transforming growth factor-β (TGF-β) signaling pathways (50–52). Several radiation-related miRNAs have been identified that contribute to the radiosensitivity of cancer cells by modulating the radiation-response signaling pathway (51,52). Since miRNAs are generally slightly repressed by their target genes, the alteration of an individual miRNA is insufficient for accomplishing a biological function. Previous studies have introduced the concept of miRNA regulatory modules (MRMs), which potentially serve as a model for understanding the detailed influences of miRNAs in cellular biological functions (53–55). Therefore, in the present study, we were particularly interested in the consequences of changes in a group of radiation-induced miRNAs in prostate cancer. Our data indicated that targets of the co-expressed miRNAs were enriched in a radiation-related signaling pathway, suggesting that they co-modulated an abundance of target genes in the same pathway (Table III).

miRNAs regulate various factors in radiation-related biological pathways and may affect the radiosensitivity of tumor cells (51). Radiation-response miRNAs have been identified in prostate cancer by using a microarray approach (13–15). By comparing these data, we identified known and unknown radiation-response miRNAs in prostate cancer by using an NGS approach. Li *et al* reported that the expression levels of miR-9, miR-22 and miR-30a decreased in radiation-treated PC3 cells (14). Radiation reduced the expression level of an miR-17-92a cluster and the let-7 family in prostate cancer (15). In the present study, we also identified radiation-response miRNAs that had been reported in other types of cancer but not in prostate cancer, such as miR-25, miR-15a, miR-30d, miR-125a, miR-221 and miR-342 (21,56–63). In addition, we identified a group of radiation-response miRNAs that have not been reported in any type of cancer. The pathway-enrichment analysis revealed that their targets are frequently enriched in the radiation-response signaling pathway.

In summary, in the present study, we thoroughly investigated radiation-response miRNAs, which may be involved in the radiosensitivity of prostate cancer, by modulating radiation-related signaling pathways using an NGS approach. These miRNA candidates may be effective targets for improving the efficacy of radiation treatment in future prostate cancer therapy. In addition, we observed that 3′ end modifications and the -5p/-3p arm selection of miRNAs were altered in prostate cancer after radiation treatment. These finding require further research.

## Figures and Tables

**Table I:** Summary of sequence reads and the detected miRNAs.

Library	Clean read (n)	miRNA read (%)	pre-miRNA (n)	miRNA (n)
P_C	9,482,400	80.49	693	916
P_5	9,748,570	79.75	687	915
P_15	9,589,440	80.35	712	933
P_40	10,589,934	80.86	739	964

P_C, P_5, P_15 and P_40 are prostate cancer cell lines with different radiation treatment. By mapping the clean sequence reads back to pre-miRNAs, we can quantify how many pre-miRNAs and mature miRNAs were detected.

**Table II:** miRNAs with altered expression in response to radiation in PC3 cells using next-generation sequencing.

	0 h	5 h	15 h	40 h	Expression data for TCGA
Upregulation^a^					
hsa-miR-9-5p	1	2.82	0.90	2.63	
hsa-miR-22-3p	1	1.60	2.85	2.73	
hsa-miR-25-3p	1	1.54	1.39	2.33	Upregulation^d^
hsa-miR-30a-5p	1	2.81	2.43	2.33	Upregulation^c^
hsa-miR-550a-3p	1	1.88	1.46	2.09	Upregulation^d^
hsa-miR-548h-5p	1	0.50	0.44	2.56	
Downregulation^b^					
hsa-let-7c	1	0.93	0.78	0.45	
hsa-let-7d-5p	1	0.30	0.11	0.40	Upregulation^d^
hsa-let-7e-5p	1	0.58	0.55	0.40	
hsa-miR-15a-5p	1	0.78	0.67	0.45	Upregulation^d^
hsa-miR-17-3p	1	0.52	0.49	0.47	Upregulation^d^
hsa-miR-30d-3p	1	0.92	0.75	0.41	Upregulation^d^
hsa-miR-92a-5p	1	0.65	0.52	0.50	Upregulation^d^
hsa-miR-125a-3p	1	0.42	0.42	0.32	
hsa-miR-197-3p	1	0.77	0.79	0.44	Upregulation^d^
hsa-miR-221-5p	1	0.59	0.31	0.44	Downregulation^d^
hsa-miR-320b	1	0.65	0.41	0.32	Upregulation^d^
hsa-miR-342-5p	1	0.59	0.63	0.47	Upregulation^d^
hsa-miR-361-3p	1	0.45	0.53	0.40	Upregulation^d^
hsa-miR-374a-5p	1	1.04	0.93	0.47	
hsa-miR-501-3p	1	0.77	0.48	0.45	Upregulation^d^
hsa-miR-671-3p	1	0.77	0.62	0.41	Upregulation^d^

a Expression levels of miRNA were inducted >2-fold change after PC3 radiation treatment with 10 Gy for 40 h.

b Expression levels of miRNA were repressed >2-fold change after PC3 radiation treatment with 10 Gy for 40 h.

c, d The difference was indicated to be significant with p-value less than 0.01 or 0.001.

**Table III:** The enriched pathways of radiation-induced miRNA target genes.

microRNA	Cancer-relative pathway (FDR<0.05)
Upregulation	
hsa-miR-9-5p	Focal adhesion, pathways in cancer, **ErbB signaling pathway**, **MAPK signaling pathway**, prostate cancer
hsa-miR-22-3p	Chronic myeloid leukemia, **MAPK signaling pathway**, **ErbB signaling pathway**, pathways in cancer, glioma, prostate cancer, **phosphatidylinositol signaling system**, colorectal cancer
hsa-miR-25-3p	N.D
hsa-miR-30a-5p	N.D
hsa-miR-550a-3p	N.D
hsa-miR-548h-5p	N.D
Downregulation	
hsa-let-7c	**MAPK signaling pathway**, pathways in cancer, **p53 signaling pathway**, melanoma, chronic myeloid leukemia, glioma, pancreatic cancer, focal adhesion, small cell lung cancer, bladder cancer, prostate cancer
hsa-let-7d-5p	**MAPK signaling pathway**, pathways in cancer, **p53 signaling pathway**, melanoma, chronic myeloid leukemia, glioma, pancreatic cancer, focal adhesion, small cell lung cancer, bladder cancer, **prostate cancer**
hsa-let-7e-5p	**MAPK signaling pathway**, pathways in cancer, **p53 signaling pathway**, melanoma, chronic myeloid leukemia, glioma, pancreatic cancer, focal adhesion, small cell lung cancer, bladder cancer, prostate cancer
hsa-miR-15a-5p	Pathways in cancer, regulation of actin cytoskeleton, renal cell carcinoma, **MAPK signaling pathway**, focal adhesion, melanoma, **prostate cancer**, **Wnt signaling pathway**, **p53 signaling pathway**, **mTOR signaling pathway**, non-small cell lung cancer, pancreatic cancer, cell cycle
hsa-miR-17-3p	**MAPK signaling pathway**, pathways in cancer, chronic myeloid leukemia, pancreatic cancer, melanoma, bladder cancer, **TGF-β signaling pathway**, **prostate cancer**, **mTOR signaling pathway**, non-small cell lung cancer, renal cell carcinoma, **cell cycle**, **p53 signaling pathway**
hsa-miR-30d-3p	N.D
hsa-miR-92a-5p	N.D
hsa-miR-125a-3p	**MAPK signaling pathway**, adherens junction, pancreatic cancer, **TGF-β signaling pathway**
hsa-miR-197-3p	N.D
hsa-miR-221-5p	**Wnt signaling pathway**, **ErbB signaling pathway** hsa-miR-320b Chronic myeloid leukemia, non-small cell lung cancer, glioma, pathways in cancer, focal adhesion, pancreatic cancer, melanoma, **ErbB signaling pathway**, colorectal cancer, **TGF-β signaling pathway**, prostate cancer, **MAPK signaling pathway**, **mTOR signaling pathway**
hsa-miR-342-5p	N.D
hsa-miR-361-3p	Pathways in cancer, **mTOR signaling pathway**, melanogenesis, renal cell carcinoma, **nucleotide excision repair**
hsa-miR-374a-5p	Pathways in cancer, prostate cancer, **TGF-β signaling pathway**, endometrial cancer, non-small cell lung cancer, basal cell carcinoma, **MAPK signaling pathway**
hsa-miR-501-3p	N.D
hsa-miR-671-3p	N.D

**Table IV:** The enriched pathways of radiation-upregulated miRNA target genes.

Pathway Id	Pathway name	FDR
Path:04360	Axon guidance	7.88E-08
Path:04722	Neurotrophin signaling pathway	1.11E-07
Path:04010	**MAPK signaling pathway**	1.11E-07
Path:05200	Pathways in cancer	3.25E-07
Path:04012	**ErbB signaling pathway**	3.14E-06
Path:04120	Ubiquitin mediated proteolysis	8.82E-06
Path:04144	Endocytosis	1.24E-05
Path:04520	Adherens junction	5.86E-05
Path:05412	Arrhythmogenic right ventricular cardiomyopathy (ARVC)	7.26E-05
Path:04510	Focal adhesion	0.000105
Path:04916	Melanogenesis	0.000245
Path:05220	Chronic myeloid leukemia	0.000245
Path:04810	Regulation of actin cytoskeleton	0.000261
Path:05214	Glioma	0.000261
Path:05414	Dilated cardiomyopathy	0.000268
Path:04910	Insulin signaling pathway	0.000282
Path:04720	Long-term potentiation	0.000294
Path:04070	**Phosphatidylinositol signaling system**	0.000294
Path:05410	Hypertrophic cardiomyopathy (HCM)	0.000802
Path:05100	Bacterial invasion of epithelial cells	0.001018
Path:05215	**Prostate cancer**	0.001096
Path:04310	**Wnt signaling pathway**	0.001153
Path:04914	Progesterone-mediated oocyte maturation	0.002548
Path:05211	Renal cell carcinoma	0.003135
Path:04920	Adipocytokine signaling pathway	0.003902
Path:04350	**TGF-β signaling pathway**	0.003902
Path:04666	Fc γ R-mediated phagocytosis	0.004314
Path:04141	Protein processing in endoplasmic reticulum	0.005738
Path:05210	Colorectal cancer	0.005747
Path:04130	SNARE interactions in vesicular transport	0.007624
Path:05216	Thyroid cancer	0.008679
Path:00562	Inositol phosphate metabolism	0.008679
Path:00532	Glycosaminoglycan biosynthesis-chondroitin sulfate	0.008679
Path:05014	Amyotrophic lateral sclerosis (ALS)	0.008679
Path:05212	Pancreatic cancer	0.008679
Path:04020	Calcium signaling pathway	0.008679
Path:05218	Melanoma	0.010147
Path:04930	Type II diabetes mellitus	0.018227
Path:04962	Vasopressin-regulated water reabsorption	0.018227
Path:04530	Tight junction	0.019289
Path:04512	ECM-receptor interaction	0.019289
Path:04912	GnRH signaling pathway	0.019289
Path:05223	Non-small cell lung cancer	0.023349
Path:05222	Small cell lung cancer	0.032425
Path:05213	Endometrial cancer	0.035464
Path:04662	B cell receptor signaling pathway	0.035464
Path:05131	Shigellosis	0.035464
Path:05221	Acute myeloid leukemia	0.03804
Path:04540	Gap junction	0.041068
Path:00250	Alanine, aspartate and glutamate metabolism	0.045237
Path:04114	Oocyte meiosis	0.049286

**Table V:** The enriched pathways of radiation-downregulated miRNA target genes.

Pathway Id	Pathway name	FDR
Path:04010	**MAPK signaling pathway**	0
Path:04360	Axon guidance	0
Path:05200	**Pathways in cancer**	0
Path:04722	Neurotrophin signaling pathway	1.18E-12
Path:04310	**Wnt signaling pathway**	1.50E-10
Path:04510	Focal adhesion	4.27E-10
Path:04144	Endocytosis	1.40E-09
Path:04810	Regulation of actin cytoskeleton	2.34E-09
Path:05215	**Prostate cancer**	3.58E-09
Path:05211	Renal cell carcinoma	3.58E-09
Path:04720	Long-term potentiation	1.14E-08
Path:05220	Chronic myeloid leukemia	3.41E-08
Path:04120	Ubiquitin mediated proteolysis	7.40E-08
Path:04020	**Calcium signaling pathway**	1.10E-07
Path:05212	Pancreatic cancer	1.57E-07
Path:05214	Glioma	1.59E-07
Path:05218	Melanoma	2.36E-07
Path:05223	Non-small cell lung cancer	3.64E-07
Path:04916	Melanogenesis	4.67E-07
Path:04520	Adherens junction	4.67E-07
Path:04910	Insulin signaling pathway	6.79E-07
Path:04350	**TGF-β signaling pathway**	7.72E-07
Path:05210	Colorectal cancer	2.50E-06
Path:04012	**ErbB signaling pathway**	3.89E-06
Path:05222	Small cell lung cancer	3.20E-05
Path:04730	Long-term depression	5.13E-05
Path:05221	Acute myeloid leukemia	7.50E-05
Path:04150	**mTOR signaling pathway**	8.68E-05
Path:05213	Endometrial cancer	8.68E-05
Path:05217	Basal cell carcinoma	9.44E-05
Path:04710	Circadian rhythm-mammal	9.64E-05
Path:04070	Phosphatidylinositol signaling system	9.64E-05
Path:04540	Gap junction	9.83E-05
Path:04115	**p53 signaling pathway**	0.000113
Path:04141	Protein processing in endoplasmic reticulum	0.000137
Path:04114	Oocyte meiosis	0.000196
Path:05014	Amyotrophic lateral sclerosis (ALS)	0.000353
Path:04970	Salivary secretion	0.000414
Path:04660	T cell receptor signaling pathway	0.000449
Path:04666	Fc γ R-mediated phagocytosis	0.000526
Path:04512	ECM-receptor interaction	0.000526
Path:05142	Chagas disease	0.000563
Path:04062	Chemokine signaling pathway	0.000812
Path:04210	**Apoptosis**	0.000844
Path:04914	Progesterone-mediated oocyte maturation	0.001465
Path:04110	**Cell cycle**	0.001527
Path:04662	B cell receptor signaling pathway	0.00169
Path:04530	Tight junction	0.001692
Path:04930	Type II diabetes mellitus	0.002128
Path:05412	Arrhythmogenic right ventricular cardiomyopathy (ARVC)	0.002128
Path:04920	Adipocytokine signaling pathway	0.002184
Path:04664	Fc ɛ RI signaling pathway	0.002621
Path:04960	Aldosterone-regulated sodium reabsorption	0.002802
Path:04912	GnRH signaling pathway	0.002955
Path:04320	Dorso-ventral axis formation	0.00373
Path:05219	Bladder cancer	0.00373
Path:04340	Hedgehog signaling pathway	0.005218
Path:04971	Gastric acid secretion	0.005633
Path:04130	SNARE interactions in vesicular transport	0.006926
Path:00532	Glycosaminoglycan biosynthesis-chondroitin sulfate	0.006926
Path:05131	Shigellosis	0.008185
Path:05160	Hepatitis C	0.008185
Path:04630	Jak-STAT signaling pathway	0.008368
Path:05410	Hypertrophic cardiomyopathy (HCM)	0.009939
Path:05414	Dilated cardiomyopathy	0.010733
Path:00534	Glycosaminoglycan biosynthesis-heparan sulfate	0.011359
Path:04670	Leukocyte transendothelial migration	0.011852
Path:04370	VEGF signaling pathway	0.013265
Path:00562	Inositol phosphate metabolism	0.013625
Path:04270	Vascular smooth muscle contraction	0.014557
Path:00512	O-Glycan biosynthesis	0.016472
Path:04330	Notch signaling pathway	0.026769
Path:04142	Lysosome	0.038882
Path:00533	Glycosaminoglycan biosynthesis-keratan sulfate	0.038882
Path:05145	Toxoplasmosis	0.048785
